# Mochi-Induced Duodenal Obstruction and Ulcers: A Case Report

**DOI:** 10.7759/cureus.84264

**Published:** 2025-05-17

**Authors:** Shogo Shirota, Nobuyasu Hirai, Hajime Hanno

**Affiliations:** 1 General Medicine, Osaka Medical and Pharmaceutical University Hospital, Takatsuki, JPN; 2 Internal Medicine, Tesseikai Neurologial Surgery Hospital, Shijonawate, JPN; 3 Gastroenterology, Tesseikai Neurologial Surgery Hospital, Shijonawate, JPN

**Keywords:** duodenal bulb obstruction, duodenal ulcer disease, gastric ulcer disease, mochi, rice cake

## Abstract

Mochi, or rice cake, is a highly adhesive food, and it is known to cause small bowel obstruction and gastric ulcers. However, reports of duodenal involvement due to mochi are rare. We report a case of duodenal obstruction and ulcers caused by mochi. A 64-year-old man with a history of diabetes mellitus presented with abdominal pain and vomiting after consuming mochi. Computed tomography (CT) of the abdomen revealed multiple high-density materials in the duodenum and stomach. Endoscopy identified multiple ulcers in the duodenum and stomach, along with mochi impaction in the duodenum. The obstruction was successfully relieved by endoscopic fragmentation. In patients with diabetes mellitus, impaired gastrointestinal motility may contribute to duodenal obstruction by mochi. When high-intensity material is observed in the stomach or duodenum on CT, confirming dietary intake is crucial for an accurate diagnosis.

## Introduction

Mochi, a traditional Japanese rice cake made from processed glutinous rice, is a highly viscous food primarily consumed in East Asia, particularly during the New Year in Japan. Due to its high stickiness, mochi is known to cause airway obstruction, gastrointestinal obstruction, and peptic ulcers [1,2]. It has even been reported to account for 10% of cardiopulmonary arrests caused by choking [1], making it a hazardous food for elderly individuals who are prone to aspiration.

Regarding gastrointestinal complications related to mochi ingestion, a Japanese report noted that nearly half of the cases occurred in January, with distal small bowel obstruction (jejunum or ileum) being the most frequently observed complication [3]. There have also been reports of gastric ulcers, small intestinal ulcers, and even perforation [3,4]. While many cases are managed conservatively, some require surgical intervention [4,5]. Other reported complications, though rare, include esophageal burns, esophageal ulcers, esophageal obstruction, duodenal ulcers, and duodenal obstruction [2,6-8]. In particular, duodenal ulcer and obstruction due to mochi have only been reported in two Japanese-language print publications [9,10].

Food-induced intestinal obstruction itself is uncommon, accounting for only 0.57% to 4.0% of all cases of intestinal obstruction [11,12]. Duodenal obstruction is even rarer, with no cases reported among 31 patients of food-related intestinal obstruction in one study [13]. Besides mochi, other reported food items causing duodenal obstruction include mushrooms and apricot pits [14,15].

We hereby report a case of duodenal obstruction and ulcers caused by mochi in a patient with diabetes mellitus.

## Case presentation

A 64-year-old man presented in early January with a two-day history of epigastric pain. The intermittent pain worsened after dinner the previous night, and he experienced three episodes of vomiting on the day of presentation. He had a nine-year history of diabetes mellitus and was being treated with metformin, dapagliflozin, and sitagliptin, with a recent hemoglobin A1c (HbA1c) level of approximately 7.5%. He also had a history of a gastric ulcer several years prior, for which *Helicobacter pylori* eradication therapy had been completed. He was not taking any nonsteroidal anti-inflammatory drugs or antiplatelet agents.

Physical examination revealed a blood pressure of 154/71 mmHg, a pulse rate of 87/min, and a body temperature of 36.6°C. The abdomen was flat and soft, with normal bowel sounds. There was tenderness at the midline of the upper abdomen without rebound. Laboratory findings revealed a white blood cell count of 17,080/μL, hemoglobin level of 18.9 g/dL, platelet count of 300,000/μL, serum creatinine of 0.88 mg/dL, blood urea nitrogen (BUN) of 19.8 mg/dL, amylase of 67 U/L, blood glucose of 206 mg/dL, and C-reactive protein (CRP) level of 0.26 mg/dL, indicating an inflammatory response and dehydration (Table 1).

**Table 1:** Laboratory findings on admission BUN: blood urea nitrogen; Cre: creatinine; CRP: C-reactive protein

Laboratory parameters	Results	Reference range
White blood cell	17,080	3,300-8,600 /µL
Hemoglobin	18.9	13.7-16.8 g/dL
Platelet	30	15.8-34.8 ×10^4^/µL
BUN	19.8	8.0-20.0 mg/dL
Cre	0.88	0.65-1.07 mg/dL
Amylase	67	44-132 U/L
Glucose	206	70-110 mg/dL
CRP	0.26	0.00-0.14 mg/dL

**Figure 1:**
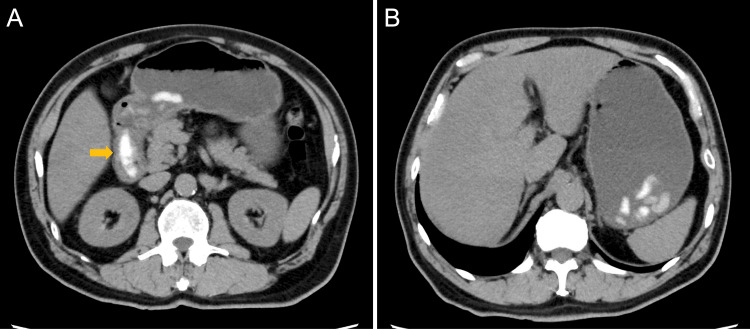
Computed tomography of the abdomen CT of the abdomen showing a high-density content measuring approximately 4 cm in length in the duodenal bulb (A, arrow), along with multiple high-density contents in the stomach and gastric distension (B).

Computed tomography (CT) of the abdomen revealed a high-density content measuring approximately 4 cm in length in the duodenal bulb, along with multiple high-density contents in the stomach and gastric distension (Figure 1).

Further history taking revealed that the patient had consumed several pieces of mochi five days and one day before presentation. Based on these findings, duodenal obstruction due to mochi was suspected, and decompression along with endoscopic removal was planned.

**Figure 2:**
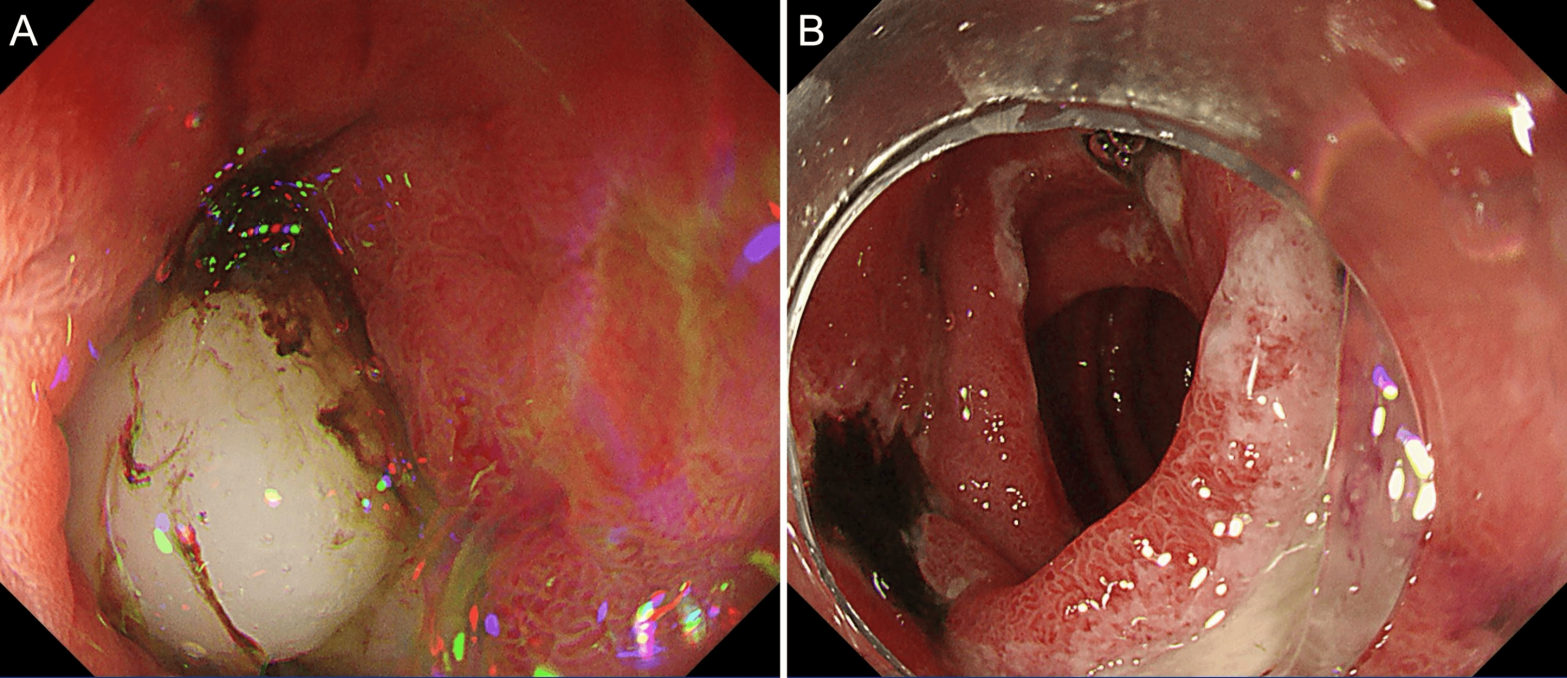
Endoscopy of the duodenum Endoscopic images showing impacted mochi (A) and A1-stage ulcers (early active stage) with adherent hematin (B) in the duodenal bulb.

A nasogastric tube was inserted, and aspiration of the gastric contents relieved the abdominal pain. Upper gastrointestinal endoscopy revealed an impacted mochi and A1-stage ulcers (early active stage, characterized by a thick mucus coating and marginal elevation due to mucosal edema) with adherent hematin in the duodenal bulb and superior duodenal angle (Figure 2) along with numerous pieces of mochi in the stomach.

**Figure 3:**
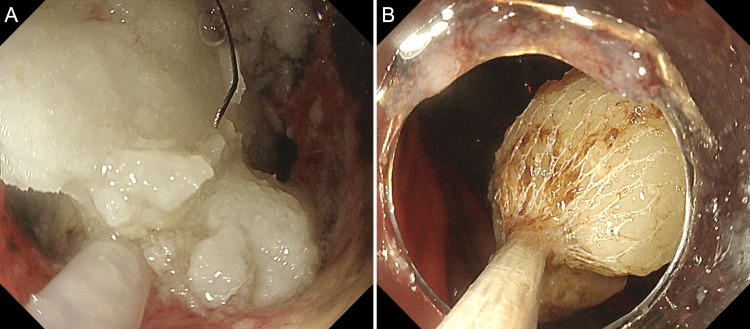
Endoscopic removal of the mochi Endoscopic images showing the snare cutting the mochi (A) and fragments retrieved with a net (B).

The obstruction was relieved using a snare to cut the mochi, and as much of the mochi as possible was removed with a retrieval net (Figure 3).

**Figure 4:**
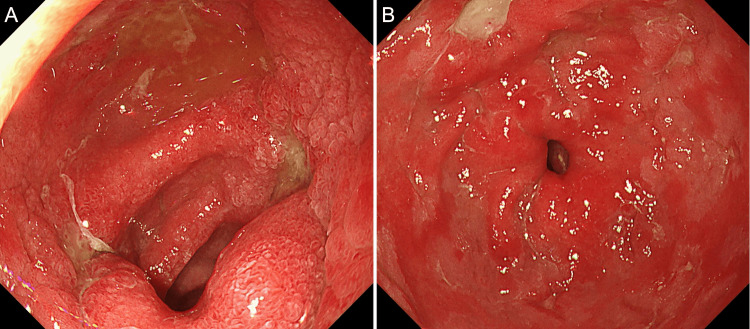
Endoscopic findings on day 4 Follow-up endoscopy showing A2-stage ulcers (later active stage) in the duodenal bulb (A) and an ulcer in the lesser curvature of the antrum along with erosive gastritis in the antrum (B).

After the procedure, the patient was admitted to the hospital for monitoring to check for any ulcer perforation or recurrence of the obstruction, and intravenous administration of omeprazole was initiated. On day 4, follow-up endoscopy revealed no residual mochi remained. A2-stage ulcers (later active stage, showing reduced edema, sharply demarcated margins, often with whitish coating on the ulcer base) were found in the angular region, antrum, duodenal bulb, and superior duodenal angle, along with erosive gastritis in the antrum (Figure 4). No tumors or strictures were observed in the duodenum.

The patient was discharged on day 5 with a prescription for vonoprazan. At the follow-up visit conducted by his primary care physician two months post-discharge, the patient reported no recurrence of symptoms.

## Discussion

This case underscores several important findings. Duodenal obstruction can be caused by mochi, especially in patients with diabetes. When hyperdense contents in the duodenum and gastric distension are observed on CT, reviewing the patient’s dietary history may aid in diagnosis. Additionally, endoscopic treatment was effective in resolving duodenal obstruction caused by mochi.

The site of mochi impaction in this case was the duodenum, which has been rarely reported in the literature [9,10]. Patients with diabetes mellitus are prone to gastrointestinal motility dysfunction [16], which may have resulted in mochi stagnation. In diabetic patients, gastroparesis is well recognized, but delayed transit can also occur throughout the gastrointestinal tract, including the duodenum. Prolonged retention of mochi in the duodenum, combined with its stickiness and hardness [2], may have contributed to mucosal injury and ulcer formation. Subsequent mucosal thickening could have led to luminal narrowing, thereby increasing the risk of obstruction. Another risk factor for intestinal obstruction due to mochi is inadequate chewing, often because of issues such as dentures, leading to the ingestion of large pieces [2].

The diagnosis of mochi-induced duodenal obstruction is based on symptoms such as acute abdominal pain and vomiting, along with CT findings of high-density material in the duodenum and gastric distention. Gastric distension is nonspecific and can occur simply due to large food or fluid intake. Therefore, it is important to assess for any stenosis or obstruction beyond the gastric outlet or duodenum. Other causes of duodenal obstruction include malignancies, inflammatory diseases (e.g., pancreatitis, duodenitis, and retroperitoneal fibrosis), and mechanical causes (e.g., superior mesenteric artery syndrome, foreign bodies, gallstones, and pancreatic cysts) [10,14,15,17,18]. Foods that appear high-density on CT include carbohydrate- or starch-rich foods such as rice, pasta, and mochi [19,20]. If high-density material is observed in the stomach or duodenum on CT, confirming recent dietary intake, including mochi, is crucial for making an accurate diagnosis, as demonstrated in our case.

Upper gastrointestinal endoscopy was effective in relieving duodenal obstruction caused by mochi. Previous reports have described the extraction of mochi in the duodenum using basket forceps [9,10]. In this case, since the mochi was large, it was finely cut using a snare and removed. Reports of small bowel obstruction caused by mochi have described improvement with bowel rest [20]. However, objects larger than 2 cm are more likely to become lodged in the gastrointestinal tract and remain impacted [2]. Therefore, in cases of upper gastrointestinal obstruction due to a large piece of mochi, prompt endoscopic treatment is recommended.

## Conclusions

This case highlights that mochi, a traditional Japanese food with high viscosity, can cause duodenal obstruction and ulceration. Although such cases are rare, they should be considered in the differential diagnosis when patients, particularly those with diabetes or impaired gastrointestinal motility, present with acute abdominal symptoms and high-density material is observed in the duodenum on CT imaging. Detailed dietary history plays a crucial role in identifying food-related causes, and early endoscopic intervention can be both diagnostic and therapeutic. Clinicians should be aware of the potential for mochi-related gastrointestinal obstruction, especially during periods of increased consumption, such as the New Year, to ensure timely and appropriate management.
